# Assessing the prevalence of insomnia and its socio-behavioral determinants among school going adolescents in Bagamati Province, Nepal

**DOI:** 10.1371/journal.pgph.0004083

**Published:** 2025-01-06

**Authors:** Mahesh Sharma, Parvati Bista, Bijay Khatri, Dipak Prasad Upadhyaya, Vijay Kumar Khanal, Bhim Prasad Sapkota, Dipak Prasad Tiwari, Nilambar Jha, Dharanidhar Baral, Gyanu Nepal Gurung, Dilaram Acharya

**Affiliations:** 1 B.P. Memorial Health Institute and Research Center, Kathmandu, Nepal; 2 Ministry of Health and Population, Kathmandu, Nepal; 3 School of Public Health, B.P. Koirala Institute of Health Science, Dharan, Sunsari, Nepal; 4 Academic & Research Department, Hospital for Children, Eye, ENT, & Rehabilitation Services (CHEERS), Madhyapur Thimi, Bhaktapur, Nepal; 5 Department of Population and Quantitative Health Sciences, Case Western Reserve University, Cleveland, Ohio, United States of America; 6 Cumming School of Medicine, University of Calgary, Alberta, Canada; PLOS: Public Library of Science, UNITED STATES OF AMERICA

## Abstract

Insomnia among adolescents is a prevalent public health concern and is closely linked to suicidal tendencies, health risk behaviors, and other adverse health outcomes. This study builds on existing literature by exploring the multifaceted associations between insomnia and socio-behavioral factors, which are currently underexplored in the Nepalese context. It assesses the prevalence of insomnia and its association with socio-behavioral factors and internet addiction among adolescents in Bagmati Province, Nepal, to inform targeted public health interventions. From July to September 2022, a school-based descriptive cross-sectional study was conducted among grade 9 and 10 students (aged 13–19) using a self-administered semi-structured questionnaire. A questionnaire included the 7-item Insomnia Severity Index (ISI) for insomnia assessment and the 20-item Young’s Internet Addiction Test for evaluating internet addiction. Binary logistic regression analysis was utilized to identify factors associated with insomnia. The study identified a significant prevalence of insomnia at 24.2%. Key socio-behavioral determinants included religion [AOR 3.58; 95% CI 1.56–8.23, AOR 3.36; 95% CI 1.27–8.89], experience of a break up [AOR 1.67; 95% CI 1.10–2.55] absence of close friendships [AOR 2.62; 95% CI 1.32–5.19], exposure to bullying [AOR 1.74, 95% CI 1.12–2.70], and internet addiction [AOR 2.74; CI 1.83–4.11]. These findings highlight the complex interplay of individual and environmental factors influencing insomnia. The significant prevalence of insomnia among school-going adolescents in Bagmati Province underscores the necessity for enhancing the role of schools in health counselling that considers behavioural, social, and demographic factors. Addressing internet addiction, fostering healthy social connections, and acknowledging the impact of demographic factors like religion could enhance intervention strategies.

## Introduction

Insomnia, characterized by difficulty initiating or maintain sleep, disproportionately affects adolescents, impacting their daytime and overall well-being [1–3]. Persistent sleep restriction leads to issues such as tiredness, daytime sleepiness, clumsiness, and weight fluctuations [4]. In adolescents, insomnia serves as an indicator of psychological distress, significantly linked to suicidal tendencies, health risk behaviors, and poor health outcomes [5]. Psychological distress, including insomnia, is associated with being bullied and engaging in fights, significant issues of violence in adolescents [6, 7].

Adolescence is a crucial, vulnerable and formative time for social, sexual, psychological and emotional behaviours that are essential to maintain sound mental health [8, 9]. Insomnia disorder is also very common in adolescents. Heavy smoking, frequent alcohol and coffee intake, lack of regular exercise, poor diet, and skipping breakfast are associated with insomnia among adolescents [10]. Excessive social media use in adolescents is speculated to be a risk factor for various mental health problems, with a positive relationship observed between insomnia and internet-based social media use [11, 12]. Internet addiction disorder has been associated with similar social consequences as that of impulse control disorder and substance abuse disorders like loss of control and withdrawal symptoms [13]. Poverty, history of death of a family member, bullying, poor family discipline, bad academic history and exposure to violence are all common risk factors for mental health disorders like insomnia which explain the premature death in adolescents age group [13, 14].

In Nepal, over 10% of children and adolescents aged 5–17 years experience anxiety, and over 2% suffer from depression [15]. The prevalence of any kind of mental disorder among adolescents was 5.2% [16]. Nepal has the second highest youth (15–29 years) suicide rate in South East Asia at 25.8 per 100,000. Additionally, 10% of adolescents have attempted suicide [17]. Insomnia among adolescents has a direct contributing role in developing major depression and anxiety [18, 19]. Insomnia among young adults was linked to factors such as social media use, living conditions, age, alcohol use, religion, sexual activity, and academic performance [20, 21]. Additionally, ethnicity, tobacco use, relationships, and using the internet before sleep were also significantly associated with poor sleep quality [22]. Over 30% of the population experiences psychiatric issues, a significant treatment gap exists with only 10% having proper mental health care access, particularly among adolescents [23].

About 75.6% of the young people had reported a negative impact on their mental health, with worsening mental health being one of the five identified themes [24]. During this time, the mental health of the children and adolescents in Nepal was affected by disrupted routines, school closures, movement restrictions, uncertainty, and fear of infection for themselves and their families [17]. The COVID-19 pandemic has further complicated the landscape, with a notable increase in internet usage among adolescents, potentially heightening the risk of insomnia [25].

In developing countries like Nepal, mental health disorders such as insomnia in adolescence often go undiagnosed and ignored issue due to limited access to psychiatric services and social stigma. Moreover, these issues can lead to behavioral problems at home and school, yet they are rarely addressed by schools and families. Adolescent mental health problems pose a significant public health challenge and hinder progress towards achieving sustainable development Goals 3.4 and 3.5 [26].

The Government of Nepal has integrated adolescent mental health and sleep problems into a general adolescent health framework, such as the revised National Adolescent Health and Development Strategy (NAHDS) [27]. The National Mental Health Strategy and Action Plan 2020 addresses adolescent mental health by integrating drug abuse, and mental and psycho-social health into adolescent sexual and reproductive health programs [28]. The expansion of adolescent friendly health services under the Nepal Health Sector strategic plan 2023–2030 aims to provide adolescent friendly health services, which includes addressing insomnia issues as part of overall mental health care [29].

However, there is still a lack of insomnia-specific intervention, and more research is needed to explore effective targeted interventions for adolescent insomnia, as very few studies have focused on this issue in Nepal. This research explores socio-behavioral factors contributing to adolescent insomnia in Nepal to inform targeted interventions and policies. So, this study aims to bridge the knowledge gap by assessing the prevalence and predictors of insomnia among school-going adolescents in Bagmati Province, Nepal.

## Methods and materials

### Study design and settings

A school-based descriptive cross-sectional study was conducted from July to September 2022 in Bagmati Province, Nepal. This region, known for its cultural diversity, includes the districts of Makwanpur, Chitwan, Kathmandu, Kavrepalanchowk, and Sindhupalchowk.

### Study participants

Adolescents aged (13–19) years studying in grades 9 and 10 in public and private schools from ten schools.

### Study variables

Insomnia among school-going adolescents was the outcome variable of this study. Independent variables examined were various socio-demographic factors in adolescents, including age, gender, ethnicity, religion, family type, school type and parents’ education. Similarly, behavioral characteristics in adolescents encompass a history of break up, sleeping hours, food insecurity, junk food consumption in the last 7 days, number of close friends, sharing of difficult problems/worries, parents understand the adolescent problems, alcohol consumption, smoking and use of tobacco product, drug use and internet addiction. Other characteristics like physical attacks, fights, bullying, deliberate self-harm, suicidal thoughts, suicidal plans, suicidal plan and perception about school health nurse.

### Measurements

#### Insomnia Severity Index (ISI)

Insomnia in students was assessed using the 7-item Insomnia Severity Index (ISI), covering sleep onset, sleep maintenance, early awakening problems, sleep dissatisfaction, interference with daytime functioning, noticeability of sleep problems to others, and distress over the past two weeks. Each item was rated on a 5-point Likert scale (0 = no problem; 4 = very severe). Total scores range from 0 to 28, with interpretations: absence of insomnia (0–7); sub-threshold insomnia (8–14); moderate insomnia (15–21); severe insomnia (22–28). A cutoff score of 10 (86.1% sensitivity, 87.7% specificity) determined the presence or absence of insomnia [30, 31].

#### Internet addiction

Adolescent student internet addiction was measured using the 20-item Internet Addiction Test (IAT), assessing the consequences of internet use. A cutoff score of 40 categorized students as normal (less than 40 points) or problematic users (40 points and above), indicating the presence of addiction symptoms. A score of 40 and above was considered as the presence of addiction symptoms [21].

#### Anthropometric measurements

Participants’ Weight and height were measured using a digital scale and non-stretchable measuring tape, respectively. Body Mass Index (BMI) was calculated. Height, measured without shoes, was recorded to the nearest centimeter using a non-stretchable measuring tape over a rigid board. Weight was measured on a calibrated digital scale placed on a flat surface, taking one digit after the decimal, with participants stepping on it barefoot and without holding onto anything.

### Sampling

The sample size was determined by using the following formulae:

n = [(z^2^pq)/e^2^]x design effect

where, z = 1.96 at a 95% Confidence level, p = 0.39 [1], q = 0.61, e = 0.05 (margin of error), design effect = 1.5

The formula produced a sample size of 546, and with an additional 25% non-response rate,

Adjusted sample size = calculated sample size/ (1-non-response rate) = 727 and the final sample size for this study was 727.

**Fig 1:**
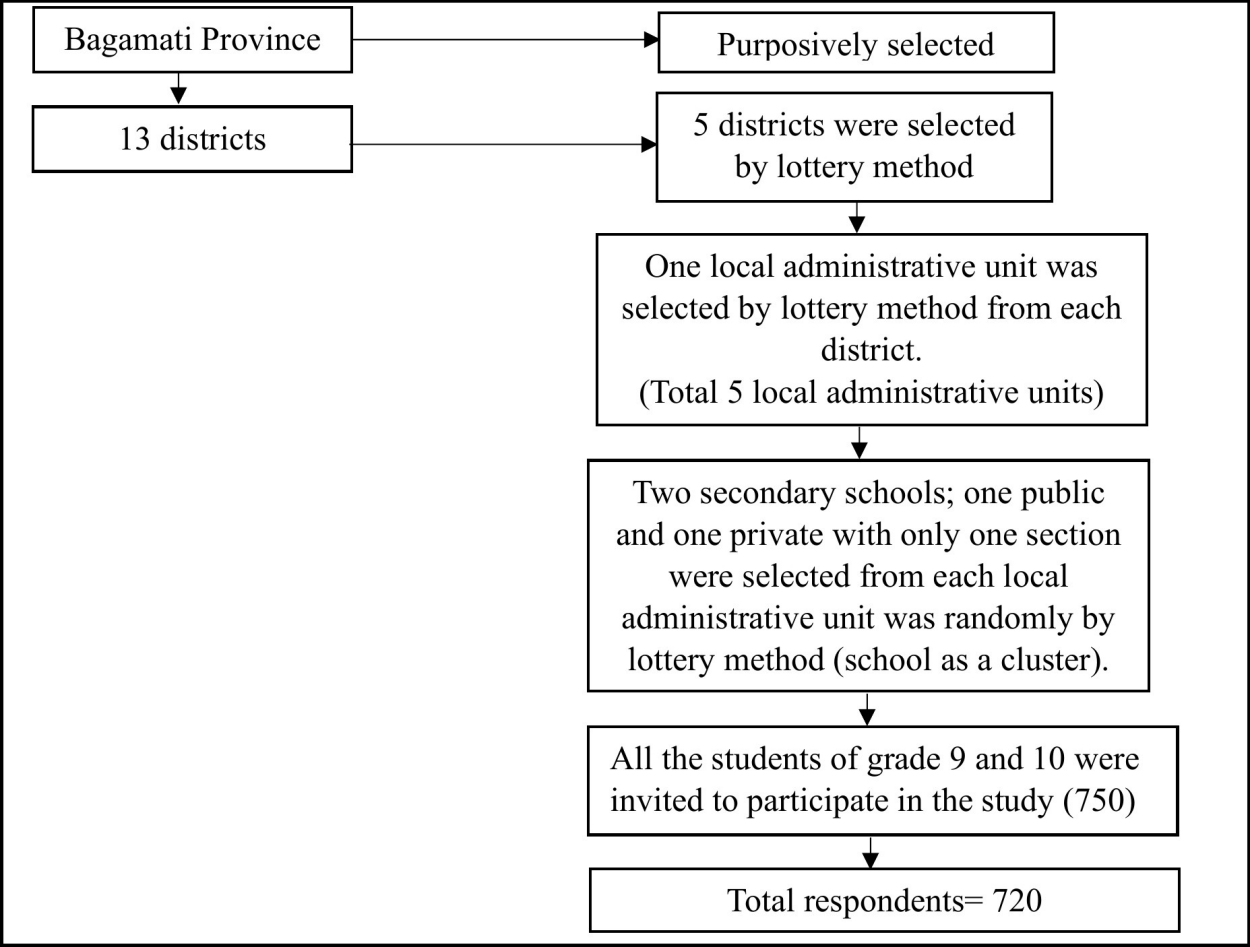
Sampling procedure.

Bagamati province was selected purposively for the study. Five districts were initially chosen at random from 13 districts of Bagamati province using a lottery method. Subsequently, one local administrative unit was selected randomly from each of the five districts. One public and one private school were chosen from each local administrative unit (school as a cluster), ensuring a diverse representation of the adolescent population. The diagram illustrating the sampling procedure is shown in Fig 1.

### Data collection tools and techniques

Data were collected using a self-administered semi-structured questionnaire, which included the Insomnia Severity Index (ISI) for assessing insomnia symptoms and Young’s Internet Addiction Test for evaluating internet addiction levels. Demographic information such as age, gender, religion, and school type were also collected. The internal consistency, measured by Cronbach’s alpha, was 0.90 for insomnia and 0.834 for internet addiction. Behavioural risk factors were evaluated using a questionnaire based on the Global School-based Health Survey (GSHS) tool by WHO. Socio-demographic and other questions were developed under the supervision of subject experts. The English-language tool was translated into Nepali, and pretested among 10% of students aged 13–19 in a school in Dharan, Sunsari of Koshi Province.

### Data processing and analysis

Data collection utilized paper-based questionnaires, with ongoing monitoring for completeness. Data were entered and validated daily, with periodic checks for accuracy and completeness Statistical analysis was conducted using SPSS, where categorical variables were summarized using frequencies and percentages, and the association between insomnia and potential predictors was analyzed using Chi-square tests and multivariate logistic regression to adjust for confounders and calculate adjusted odds ratios (AORs) with 95% confidence intervals.

### Ethical consideration

Ethical clearance was obtained from the Institutional Review Committee (IRC) of BPKIHS (Reference number: 221/078/079—IRC). The study permission was taken from the Education Development Directorate, Bagmati province, Nepal (Reference No.: 078/79–283), and respective school authorities. Participants and parents were assured of voluntary participation, with the right to withdraw at any time. Confidentiality was maintained, and gathered information was exclusively used for research purposes.

The class teachers helped distribute the informed consent forms to each student so that their parents or guardians were informed about the purpose of the study. Students below 18 years present on the data collection day without their parents and guardians signing the consent form were excluded from the study.

### Inclusivity in global research

Additional information regarding the ethical, cultural, and scientific considerations specific to inclusivity in global research is included in the Supporting Information (S1 Checklist)

### Exclusion criteria

Students absent on the day of data collection were excluded from the study.

## Results

The study surveyed 720 adolescents in grade 9 and 10, with a Mean age:14.90±1.12, where 50.8% were female). A majority (84.8%) identified as Hindu, and (58.5%) belonged to the Brahmin/Chhetri caste. About 61.0% came from nuclear families, and 69.7% attended public schools. The percentage of tobacco use and alcohol consumption among participants was found to be 5.7% and 9.8% respectively. Similarly, 36.1% of the students were addicted to internet use based on the Internet Addiction test. Nearly three-fourths (70%) of the adolescents perceived school health nurse was very important for them.

### Prevalence of insomnia and associated factors

Our findings indicate that 34.5% of participants experienced sub-threshold insomnia, while 6.1% reported moderate symptoms, and 0.8% showed signs of severe insomnia, leading to an overall prevalence rate of 24.2%. This was determined using the Insomnia Severity Index with a cut-off score >10.

Table 1 illustrates the bivariate analysis of socio-demographic factors associated with insomnia. Older adolescents (17–19 years old) have 1.63 times higher odds of developing insomnia than their younger counterparts (13–16 years old), although this was not statistically significant (p = 0.116) adolescents. Female adolescents had 1.29 times higher odds of developing insomnia compared to males. Participants belonging to Hinduism and Buddhism had significantly higher odds of developing insomnia compared to other religions (Christian and Muslim) with odds ratios of 1.78 (p = 0.0011). Similarly, adolescents studying in private schools were 1.58 times more likely to develop insomnia compared to those in public schools (p = 0.012).

**Table 1:** Bivariate analysis of socio-demographic factors associated with insomnia.

Variable	Category	Total (%) ^a^	Insomnia		COR (95% CI)	p-value
			Presence (%)	Absence (%)		
Age in years	13–16	669 (92.9)	157 (23.5)	512 (76.5)	Ref	
	17–19	51 (7.1)	17 (33.3)	34 (66.7)	1.63 (0.88–2.99)	0.116
Gender	Male	354 (49.2)	77 (21.8)	277 (78.2)	Ref	
	Female	366 (50.8)	97(26.5)	269 (73.5)	1.29 (0.92–1.83)	0.137
Ethnicity	Janajati	231 (32.1)	64 (27.7)	167 (72.3)	Ref	
	Brahmin/Chhetri	421 (58.5)	96 (22.8)	325 (77.2)	1.29 (0.89–1.87)	0.165
	Others	68 (9.4)	14 (20.6)	54 (79.4)	1.47 (0.77–2.84)	0.242
Religion	Hindu	611 (84.8)	137 (22.4)	474 (77.6)	Ref	
	Non-Hindu	109 (15.2)	37 (33.9)	72 (66.1)	1.78 (1.14–2.76)	0.011*
Family type	Nuclear	439 (60.9)	108 (24.6)	331 (75.4)	1.06 (0.75–1.51)	0.733
	Joint	281 (39.1)	66 (23.5)	215 (76.5)	Ref	
Type of school	Public	502 (69.7)	108 (21.5)	394 (78.5)	Ref	
	Private	218 (30.3)	66 (30.3)	152 (69.7)	1.58 (1.11–2.27)	0.012*
Father’s education	Illiterate	37 (5.1)	9 (24.3)	28 (75.7)	0.99 (0.46–2.14)	0.982
	Literate	683 (94.9)	165 (24.2)	518 (75.8)	Ref	
Mother’s education	Illiterate	92 (12.8)	22 (23.9)	70 (76.1)	0.98 (0.58–1.64)	0.951
	Literate	628 (87.2)	152 (24.2)	476 (75.8)	Ref	

^a^ Column Total, Ref Reference Category

* Significant, COR = Crude Odds Ratio

The analysis of behavioral factors revealed that participants with a history of break-up had 2.67 times higher odds of experiencing insomnia (p<0.001) and those who slept less than 8 hours had 1.47 times higher odds(p = 0.028). Adolescents with no close friends had a significantly higher risk of insomnia, with an odds ratio of 2.86 (p<0.001). Internet addiction also emerged as a significant factor, with addicted individuals having 2.47 times higher odds of insomnia (p<0.001) as shown in Table 2.

**Table 2:** Bivariate analysis of behavioral factors associated with insomnia.

Variable	Category^a^	Total (%)	Insomnia		COR (95% CI)	p-value
			Presence (%)	Absence (%)		
History of break up	Yes	183 (25.4)	71 (38.8)	112 (61.2)	2.67 (1.85–3.85)	<0.001*
	No	537 (74.6)	103 (19.2)	434 (80.8)	Ref	
Sleeping hours	less than 8	292 (40.6)	83 (28.4)	209 (71.6)	1.47 (1.04–2.07)	0.028*
	More than or equal to≥ 8	428 (59.4)	91 (21.3)	337 (78.7)	Ref	
Food insecurity	Never	611 (84.8)	145 (23.7)	466 (76.3)	Ref	
	Sometimes	109 (15.2)	29 (26.6)	80 (73.4)	1.16 (0.73–1.85)	0.518
Junk food consumption in last 7 days	Yes	651 (90.4)	159 (24.4)	492 (75.6)	1.16 (0.64–2.12)	0.621
	No	69 (9.6)	15 (21.7)	54 (78.3)	Ref	
Number of close friends	No any close friend	46 (6.4)	21 (45.7)	25 (54.3)	2.86 (1.56–5.25)	<0.001*
	Close friends	674 (93.6)	153 (22.7)	521 (77.3)	Ref	
Sharing difficult problems or worries	Never	153 (21.2)	42 (27.5)	111 (72.5)	Ref	
	Sometimes	447 (62.1)	103 (23.0)	344 (77.0)	1.26 (0.83–1.92)	0.272
	Most of the time	120 (16.7)	29 (24.2)	91 (75.8)	1.18 (0.68–2.05)	0.539
Parent’s understood child’s problem	Never	79 (10.9)	29 (36.7)	50 (63.3)	Ref	
	Sometimes	287 (39.9)	73 (25.4)	214 (74.6)	1.70 (1.01–2.88)	0.049*
	Most of the time	354 (49.2)	72 (20.3)	282 (79.7)	2.27 (1.34–3.84)	0.002*
Alcohol Consumption	Yes	71 (9.8)	22 (31.0)	49 (69.0)	1.46 (0.86–2.51)	0.159
	No	649 (90.2)	152 (23.4)	497 (76.6)	Ref	
Smoke cigarette and other tobacco use	Yes	41 (5.7)	18(43.9)	23 (56.1)	2.62 (1.38–4.98)	0.003*
	No	679 (94.3)	156 (23.0)	523 (77.0)	Ref	
Drug consumption	Yes	20 (2.8)	9 (45.0)	11 (55.0)	2.65 (1.08–6.51)	0.033*
	No	700 (97.2)	165 (23.6)	535 (76.4)	Ref	
Seriously injured last 12 months	Yes	343 (47.6)	70 (20.4)	273 (79.6)	0.67 (0.47–0.95)	0.025*
	No	377 (52.4)	104 (27.6)	273 (72.4)		
Internet addiction	Addiction	260 (36.1)	102 (39.2)	158 (60.8)	3.47 (2.44–4.96)	<0.001*
	Normal	460 (63.9)	72 (15.7)	388 (84.3)	Ref	

^a^ Column Total; Ref Reference Category

* Significant, COR = Crude Odds Ratio

Similarly, adolescents who had a suicidal thought and suicidal plans had increased odds of developing insomnia with 2.58 and 3.24 respectively. However, adolescent who had reported experiencing bullying were 2.8 times less likely to develop insomnia compared to their counterparts. See Table 3.

**Table 3:** Bivariate analysis of physical attacks, physical fights and other factors associated with insomnia.

Variable	Category	Total (%)	Insomnia		COR (95% CI)	p-value
			Presence (%)	Absence (%)		
Physical attacks	Yes	618 (85.8)	134 (21.7)	484 (78.3)	0.43 (0.27–0.66)	<0.001*
	No	102 (14.2)	40 (39.2)	62 (60.8)	Ref	
Physical fights	Yes	481 (66.8)	93 (19.3)	388 (80.7)	0.46 (0.33–0.66)	<0.001*
	No	239 (33.2)	81 (33.9)	158 (66.1)	Ref	
Bullying	Yes	536 (74.5)	101 (18.8)	435 (81.2)	Ref	
	No	184 (25.5)	73 (39.7)	111 (60.3)	2.83 (1.96–4.08)	<0.001*
Self-harm	Yes	518 (71.9)	106 (20.5)	412 (79.5)	0.51 (0.35–0.73)	<0.001*
	No	202 (28.1)	68 (33.7)	134 (66.3)	Ref	
Suicidal thought	Yes	132 (18.3)	53 (40.2)	79 (59.8)	2.58 (1.73–3.87)	<0.001*
	No	588 (81.7)	121 (20.6)	467 (79.4)	Ref	
Suicidal plan	Yes	86 (11.9)	40 (46.5)	46 (53.5)	3.24 (2.04–5.16)	<0.001*
	No	634 (88.1)	134 (21.1)	500 (78.9)	Ref	
Suicidal attempts	Yes	630 (87.5)	135 (21.4)	495 (78.6)	0.35 (0.22–0.56)	<0.001*
	No	90 (12.5)	39 (43.3)	51 (56.7)	Ref	
Availability of school health nurse	Yes	548 (76.1)	130 (23.7)	418 (76.3)	Ref	
	No	172 (23.9)	44 (25.6)	128 (74.4)	1.10 (0.74–1.64)	0.619
Perception about school health nurse	Very important	504 (70.0)	119 (23.6)	385 (76.4)	1.25 (0.51–3.08)	0.675
	Average	191 (26.5)	48 (25.1)	143 (74.9)	1.15 (0.45–2.94)	0.615
	Not important	25 (3.5)	7 (28.0)	18 (72.0)	Ref	

^a^ Column Total; Ref: Reference group

*: Significant at p<0.05, COR = Crude Odds Ratio

After adjusting for confounding factors, binary logistic regression analysis identified several predictors of insomnia among school-going adolescents. Adolescents belonging to Hindu and Buddhist religions had nearly three times higher risk of having insomnia as compared to other religions [AOR 3.58; 95% CI 1.56–8.23, AOR 3.36; 95% CI 1.27–8.89, respectively]. Participants with a history of breakup had a 1.67 times higher risk of having insomnia [AOR 1.67; 95% CI 1.10–2.55]. Adolescents with no close friends had two times higher risk of having insomnia [AOR 2.62; 95% CI 1.32–5.19]. Interestingly, participants with no history of bullying had almost 2-fold higher risk of having insomnia [AOR 1.74; 95% CI 1.12–2.71]. Insomnia was 2.7 times higher among those adolescents with a history of internet addiction compared to their counterparts [AOR 2.74; CI 1.83–4.11], as shown in Table 4.

**Table 4:** Binary logistics regression to determine predictor of insomnia among study participants (n = 720).

Characteristics	Category	Insomnia		COR (95% CI)	p-value	AOR (95% CI)	p-value
		Present (%)	Absent (%)				
Religion	Hindu	137 (22.4)	474 (77.6)	2.99 (1.39–6.45)	0.005*	3.58 (1.56–8.23)	0.003*
	Buddhist	24 (29.6)	57 (70.4)	2.06 (0.85–4.97)	0.109	3.36 (1.27–8.89)	0.014*
	Others	13 (46.4)	15 (53.6)	Ref		Ref	
Type of school	Public	108 (21.5)	394 (78.5)	Ref		Ref	
	Private	66 (30.3)	152 (69.7)	1.58 (1.11–2.27)	0.012*	0.99 (0.65–1.51)	0.963
History of break up	Yes	71 (38.8)	112 (61.2)	2.67 (1.85–3.85)	<0.001*	1.67 (1.10–2.55)	0.016*
	No	103 (19.2)	434 (80.8)	Ref		Ref	
Sleeping hours	<8 hours	83 (28.4)	209 (71.6)	1.47 (1.04–2.07)	0.028*	1.41 (0.96–2.55)	0.080
	≥ 8 hours	91 (21.3)	337 (78.7)	Ref		Ref	
Number of close friends	No any close friend	21 (45.7)	25 (54.3)	2.86 (1.56–5.25)	<0.001*	2.62 (1.32–5.19)	0.006*
	Close friends	153 (22.7)	521 (77.3)	Ref		Ref	
Parents understood child’s problem	Never	29 (36.7)	50 (63.3)	Ref		Ref	
	Sometimes	73 (25.4)	214 (74.6)	1.70 (1.01–2.88)	0.049*	1.18 (0.64–2.18)	0.586
	Most of the time	72 (20.3)	282 (79.7)	2.27 (1.34–3.84)	0.002*	1.18 (0.64–2.18)	0.797
Smoke cigarettes and other tobacco used	Yes	18 (43.9)	23 (56.1)	2.62 (1.38–4.98)	0.003*	1.37 (0.62–3.02)	0.430
	No	156 (23.0)	523 (77.0)	Ref		Ref	
Drug used	Yes	9 (45.0)	11 (55.0)	2.65 (1.08–6.51)	0.033*	1.53 (0.51–4.61)	0.448
	No	165 (23.6)	535 (76.4)	Ref		Ref	
Seriously injured in the last 12 months	Yes	70 (20.4)	273 (79.6)	0.67 (0.47–0.95)	0.025*	0.94 (0.63–1.39)	0.747
	No	104 (27.6)	273 (72.4)	Ref		Ref	
Internet addiction	Addiction	102 (39.2)	158 (60.8)	3.47 (2.44–4.96)	<0.001*	2.74 (1.83–4.11)	<0.001*
	Normal	72 (15.7)	388 (84.3)	Ref		Ref	
Physical attacks	Yes	134 (21.7)	484 (78.3)	0.43 (0.27–0.66)	<0.001*	0.71 (0.42–1.21)	0.211
	No	40 (39.2)	62 (60.8)	Ref		Ref	
Physical fights	Yes	93 (19.3)	388 (80.7)	0.46 (0.33–0.66)	<0.001*	1.21 (0.77–1.87)	0.413
	No	81 (33.9)	158 (66.1)	Ref		Ref	
Bullying	Yes	101 (18.8)	435 (81.2)	Ref		Ref	
	No	73 (39.7)	111 (60.3)	2.83 (1.96–4.08)	<0.001*	1.74 (1.12–2.71)	0.013*
Self-harm	Yes	106 (20.5)	412 (79.5)	0.51 (0.35–0.73)	<0.001*	0.87 (0.57–1.34)	0.538
	No	68 (33.7)	134 (66.3)	Ref		Ref	
Suicidal thought	Yes	53 (40.2)	79 (59.8)	2.58 (1.73–3.87)	<0.001*	1.22 (0.71–2.12)	0.478
	No	121 (20.6)	467 (79.4)	Ref		Ref	
Suicidal plan	Yes	40 (46.5)	46 (53.5)	3.24 (2.04–5.16)	<0.001*	1.47 (0.74–2.92)	0.273
	No	134 (21.1)	500 (78.9)	Ref		Ref	
Suicidal attempts	Yes	135 (21.4)	495 (78.6)	0.35 (0.22–0.56)	<0.001*	0.77 (0.38–1.52)	0.448
	No	39 (43.3)	51 (56.7)	Ref		Ref	

Ref: Reference group

*: Significant at p<0.05, COR = Crude Odds Ratio, AOR = Adjusted Odds Ratio

However, factors such as type of school, sleep duration, parental understanding the child’s problems, cigarette or tobacco use, drug use, serious injury, physical attacks, physical fights, self-harm, suicidal thoughts, plans and attempts were not significantly associated adolescent insomnia.

## Discussion

This study found a significant prevalence of insomnia among participants to be 24.2%. This prevalence was higher compared to studies conducted in Portugal [32] and Detroit [33] but lower than a study in Kathmandu Valley[20] and Karnataka, India [34]. Such variations likely reflect the complex interplay of socio-economic cultural and environmental factors unique to each study site. The relatively low prevalence of insomnia in this study may be attributed to the presence of a school health nurse in 76% of the schools and the emphasis placed by 70% of the respondents on the importance of school health nurses. These factors might play a role in supporting and addressing the mental health and well-being of the students.

A notable finding of our research is the higher prevalence of insomnia among female adolescents which resonates with findings from a previous study in Karnataka, India [34, 35]. The higher prevalence in girls may be linked to a greater prevalence of self-harm on the internet, particularly among girls possibly associated with the onset of menses [36]. Moreover, the study identified a significant association between internet addiction and insomnia, underscoring the pervasive impact of digital technologies on adolescent health. This link could be attributed to hormonal changes during menstruation and the higher susceptibility of girls to mood disorders like anxiety and depression, which in turn increase the risk of sleeping problems [33].

The study found that adolescents practicing Hindu and Buddhist religions had a higher risk of insomnia compared to other religions. This finding is similar to the study done in undergraduate students in Nepal where non-Hindu students experience better sleep quality in comparison to Hindu Students [21, 22]. Varied religious and cultural practices within the religious communities, might impact adolescent lifestyle, potentially leading to sleep disorders. This finding suggests the importance of exploring contextual and religious factors through conducting in-depth sociological and anthropological research, as well as the evolving technological landscape, to better understand the relationship between religion and insomnia within the target population.

The study found that insomnia was higher among adolescents who didn’t have any close friends compared to those having friends which was similar as reported by Hebbar [34]. Similarly, another study from Nepal also demonstrated a similar significant association between friends and insomnia [10]. Lack of close friendships can contribute to feelings of depression, which was strongly associated with insomnia. Depression can disrupt sleep patterns and lead to both difficulties in falling asleep and early morning awakenings [18, 37, 38].

Studies from Norway and Shakoor found a strong positive link between bullying and sleep issues, particularly among adolescents [39–41]. However, our study’s findings contrasted, possibly due to socio-cultural, age, and educational differences. Additionally, the adolescent who experiences no bullying might feel isolated and face emotional or psychological stressors that affect their sleep. This highlights the need to explore these factors to better understand the in-depth relationship between bullying and insomnia.

Adolescents with borderline and possible internet addiction were more likely to experience insomnia compared to no internet addiction which is similar to our study [42]. Similarly, our finding is supported by previous studies conducted in China [43, 44]. As the COVID-19 pandemic rages on, the internet continues to provide a lifeline. It’s more important than ever that people can access a suitable device–with sufficient data and speeds–as businesses, schools and the public are forced to move their services online [25].

The cross-sectional nature of this study limits our capacity to infer causality between insomnia and identified factors. Moreover, since the study relied on self-reported data, it is subjected to potential recall bias and response bias. Our study didn’t consider environmental factors that might influence insomnia. Additionally, it can be generalized to the adolescent population in Bagamati Province rather than the whole Nepal due to diverse socio economic and socio-cultural factors, as well as differences in accessibility to health services. The findings underscore the need for improved sociological and anthropological study designs, larger-scale investigations and broader geographical coverage to gain a clearer understanding of the prevalence of sleep disorders among child and adolescent in Nepal. Such comprehensive research is crucial for informing the planning of public health interventions to address mental health issues like insomnia effectively.

## Conclusion

This study has shown the significant prevalence of insomnia among school-going adolescents in Bagmati Province, Nepal, underscoring not only the scope of the issue but also its association with a variety of socio-behavioural and demographic factors. Particularly, the links between insomnia and internet addiction, the absence of close friendships, experiences of bullying, and the influence of socio-cultural elements like religion have been highlighted.

A key recommendation is to expand the school health nurse program to promote sleep hygiene and provide targeted counseling services on identified behavioral factors, such as internet usage habits, social interactions, and experiences of bullying that contribute to insomnia in adolescents. Additionally, schools should coordinate with health sector in integrating mental health services within the existing adolescent friendly health services, in line with National Adolescent Health and Development Strategy 2018 and National Mental Health Strategy and Action Plan 2020. In light of our findings, future sociological and anthropological research should explore the longitudinal impacts of socio-behavioral factors on insomnia and other related health outcomes in adolescents.

## Supporting information

S1 Checklist PLOS’ questionnaire on inclusivity in global research. (PDF)

S1 Text Questionnaire. (PDF)
