# Supplementary material for: Assessing the prevalence of insomnia and its socio-behavioral determinants among school going adolescents in Bagamati Province, Nepal
Source: PLOS Glob Public Health. 2025 Jan 6;5(1):e0004083. doi: 10.1371/journal.pgph.0004083 (PMC11703102; doi:10.1371/journal.pgph.0004083)
Supplement: S1 Text — (PDF) [file pgph.0004083.s002.pdf]

Questionnaire for Data Collection

Insomnia among school going adolescents of Bagmati Province, Nepal

BP Koirala Institute of health sciences, Dharan, Nepal

Code no. **Date:**

School Name:

**Socio - demographic Information:**			
**SN**	**Questions**	**Response**	**Code**
1.	Age (in completed a year)	……………..	
2.	Gender	a. Male<br>b. Female<br>c. others	
3.	Ethnicity	a. Dalit<br>b. Janjati<br>c. Madhesi<br>d. Muslim<br>e. Brahmin/ Chettri<br>f. Other	
4.	Religion	………………….	
5.	Permanent Address	…………………..	
6.	Current Address	……………………	
7.	Type of Family	a. Nuclear<br>b. Joint	
8.	Number of siblings	…………………	
9.	Education (Class)	…………………	
10.	Types of school	a. Public<br>b. Private	
11.	Fathers' education	....	
12.	Fathers' occupation	-------------------	
13.	Mother's education	………………….	
14.	Mother occupation	………………….	
15.	Monthly family Income	RS………………….	
16.	History of break up	a. Yes<br>b. No	
17.	Living arrangement	a. Living with family,	

		b. Not living with family	
18.	Sleeping time	Bedtime ……..<br>Weak up ………..	
19.	Are you satisfied with your academic performance?	a. Yes<br>b. No	
20.	How much help have you received from teacher?	a. Most of the time<br>b. sometimes<br>c. Never	
21.	Disable person in your family (physical/ mental)	a. yes<br>b. No	
**Dietary Behaviors and physical activity:**			
22.	Height (cm)		
23.	Weight (kg)		
24.	During the past 30 days, how often did you go hungry because there was not enough food in your home?	a. Never<br>b. Sometimes<br>c. Most of the time	
25.	During the past 7 days, how many times per day did you usually eat vegetables, such as cauliflower, cabbage, or Brinjal, pumkin?	a. Never<br>b. Sometimes<br>c. Most of the time	
26.	During the past 7 days, how many times did you eat fruit, such as apples, oranges, mangoes, or papayas?	a. Never<br>b. Sometimes<br>c. Most of the time	
27.	During the past 7 days how many times per day did you eat Junk food (Mo.Mo., Chaumin, pizza, burger, nodules, cock, Cocacola, fanta etc.)	a. Never<br>b. Sometimes<br>c. Most of the time	
28.	During the past 7 days, on how many days were you physically active for a total of at least 60 minutes per day?	a. 0 days<br>b. 1 day<br>c. 2 days<br>d. 3 days<br>e. 4 days<br>f. 5 days<br>g. 6 days<br>h. 7 days	
29.	During this school year, were you taught in any of your classes the benefits of physical activity?	a. Yes<br>b. No<br>c. I don't know	
**Protective Factors:**			

30.	How many close friends do you have?	a. 0<br>b. 1<br>c. 2<br>d. 3 or more	
31.	how often were you able to talk to someone about difficult problems and worries?	a. Never<br>b. Sometimes<br>c. Most of the time	
32.	How often did your parents or guardians understand your problems and worries?	a. Never<br>b. Sometimes<br>c. Most of the time	
**Alcohol, smoking, drug use and sexual activity:**			
33.	Do you drink Alcohol?	a. Yes<br>b. No	
34.	Do you smoke cigarette and other tobacco used?	a. Yes<br>b. No	
35.	Do you use drugs?	a. Yes<br>b. No	
36.	During this school year, were you taught in any of your classes the problems associated with drinking alcohol, smoking cigarettes and using drug?	a. Yes<br>b. No<br>c. I do not know	
37.	During your life, with how many people have you had sexual intercourse?	a. I have never had sexual intercourse<br>b. 1 person<br>c. 2 people<br>d. more than two people	
38.	How often do you or your partner use a condom or Dhaal when you have sexual intercourse?	a. Never use<br>b. Sometimes<br>c. Every time	
39.	During this school year, were you taught in any of your classes on STI/ HIV infection or AIDS?	a. Yes<br>b. No<br>c. I don’t know	
**Unintentional Injuries, physical attacks, physical fight and Bullying:**			
40.	During the past 12 months, how many times were you seriously injured?	a. 0 time<br>b. 1 time<br>c. 2 or more times	
41.	During the past 12 months, what was the major cause of the most serious injury that happened to you?	a. I was not seriously injured during the past 12 months	

		b. I was in a motor vehicle accident or hit by a motor vehicle<br>c. I fell<br>d. Something fell on me or hit me<br>e. I was attacked or abused or was fighting with someone<br>f. I was in a fire or too near a flame or something hot<br>g. I inhaled or swallowed something bad for me<br>h. Something else caused my injury	
42.	During the past 12 months, how many times were you physically attacked?	a. 0 time<br>b. 1 time<br>c. 2 or more times	
43.	During the past 12 months, how many times were you in a physical fight?	a. 0 time<br>b. 1 time<br>c. 2 or more times	
44.	During the past 30 days, on how many days were you bullied?	a. not bullied<br>b. 1 time<br>c. 2 or more times	
45.	Place of Bullying	a. On school property<br>b. Outside the school	
46.	Types of Bullying	a. Physical<br>b. Verbal<br>c. Social<br>d. Cyber	
**Deliberated self-harm and Suicide:**			
47.	During the past 12 month, how many times deliberately harmed yourself?	a. Not harmed<br>b. 1 time<br>c. 2 or more times	
48.	During the past 12 months, did you seriously consider attempting suicide? (*Seriously think about trying to take your own life)*	a. Yes<br>b. No	
49.	During the past 12 months, did you make a plan about how you would	a. Yes	

	attempt suicide? (*Planning how (by which method, at what time, at which place) to take his/her own life)*	b. No	
50.	During the past 12 months, how many times did you attempt suicide? (*Attempting suicide means trying to take your own life.)*	a. 0 time<br>b. 1 time<br>c. 2 or more times	
51.	Has anyone in your close family (mother, father, brother, sister) ever attempted or died from suicide?	a. Yes<br>b. No	
52.	During this school year, were you taught in any of your classes regarding signs of mental health problems (anxiety, depression and suicidal behavior) and how to improve mental health?	a. Yes<br>b. No<br>c. I do not know	
**School Health service provider:**			
53.	Is there a health worker/ school health nurse service available at your school?	a. Yes<br>b. No	
54.	What do you think, the health worker / school health nurse program is necessary for the improvement of student's health?	a. Very important<br>b. Average<br>c. Not important	

Insomnia Severity Index

The Insomnia Severity Index has seven questions. The seven answers are added up to get a total score. When you have your total score, look at the 'Guidelines for Scoring/Interpretation' below to see where your sleep difficulty fits. For each question, please Circle the number that best describes your answer. Please rate the Current (i.e. Last 2 Weeks) Severity of your insomnia problem.

**S.N.**	**Insomnia Problem**	**None**	**Mild**	**Moderate**	**Severe**	**Very Severe**
1.	Difficulty falling asleep	0	1	2	3	4
2.	Difficulty staying asleep	0	1	2	3	4
3.	Problems waking up too early	0	1	2	3	4
4.	How **satisfied / Dissatisfied** are you with your **Current** sleep pattern?	Very Satisfied (0)	Satisfied (1)	Moderately Satisfied (2)	Dissatisfied (3)	Very Dissatisfied (4)
5.	How **Noticeable** to others do you think your sleep problem is in terms of impairing the quality of your life?	Not at all Noticeable (0)	A Little (1)	Somewhat (2)	Much (3)	Very Much Noticeable (4)
6.	How **Worried/Distressed** are you about your current sleep problem?	Not at all Worried (0)	A Little (1)	Somewhat (2)	Much (3)	Very Much worried (4)
7.	To what extent do you consider your sleep problem to **Interfere** with your daily functioning (e.g. daytime fatigue, mood, ability to function at work/daily chores, concentration, memory, mood, etc.) **Currently**?	Not at all interfering (0)	A Little (1)	Somewhat (2)	Much (3)	Very Much worried (4)

**S.N.**	**Internet addiction Test**	
8.	How often do you find that you stay online longer than you intended?	0 Not Applicable<br>1 Rarely<br>2 Occasionally

		3 Frequently<br>4 Often<br>5 Always
9.	How often do you neglect household chores to spend more time online?	0 Not Applicable<br>1 Rarely<br>2 Occasionally<br>3 Frequently<br>4 Often<br>5 Always
10.	How often do you prefer the excitement of the Internet to intimacy with your partner?	0 Not Applicable<br>1 Rarely<br>2 Occasionally<br>3 Frequently<br>4 Often<br>5 Always
11.	How often do you form new relationships with fellow online users?	0 Not Applicable<br>1 Rarely<br>2 Occasionally<br>3 Frequently<br>4 Often<br>5 Always
12.	How often do others in your life complain to you about the amount of time you spend online?	0 Not Applicable<br>1 Rarely<br>2 Occasionally<br>3 Frequently<br>4 Often<br>5 Always
13.	How often do your grades or school work suffer because of the amount of time you spend online?	0 Not Applicable<br>1 Rarely<br>2 Occasionally<br>3 Frequently<br>4 Often<br>5 Always
14.	How often do you check your email before something else that you need to do?	0 Not Applicable<br>1 Rarely<br>2 Occasionally<br>3 Frequently<br>4 Often<br>5 Always

15.	How often does your job performance or productivity suffer because of the Internet?	0 Not Applicable<br>1 Rarely<br>2 Occasionally<br>3 Frequently<br>4 Often<br>5 Always
16.	How often do you become defensive or secretive when anyone asks you what you do online?	0 Not Applicable<br>1 Rarely<br>2 Occasionally<br>3 Frequently<br>4 Often<br>5 Always
17.	How often do you block out disturbing thoughts about your life with soothing thoughts of the Internet?	0 Not Applicable<br>1 Rarely<br>2 Occasionally<br>3 Frequently<br>4 Often<br>5 Always
18.	How often do you find yourself anticipating when you will go online again?	0 Not Applicable<br>1 Rarely<br>2 Occasionally<br>3 Frequently<br>4 Often<br>5 Always
19.	How often do you fear that life without the Internet would be boring, empty, and joyless?	0 Not Applicable<br>1 Rarely<br>2 Occasionally<br>3 Frequently<br>4 Often<br>5 Always
20.	How often do you snap, yell, or act annoyed if someone bothers you while you are online?	0 Not Applicable<br>1 Rarely<br>2 Occasionally<br>3 Frequently<br>4 Often<br>5 Always
21.	How often do you lose sleep due to being online?	0 Not Applicable<br>1 Rarely<br>2 Occasionally<br>3 Frequently

		4 Often<br>5 Always
22.	How often do you feel preoccupied with the Internet when off-line, or fantasize about being online?	0 Not Applicable<br>1 Rarely<br>2 Occasionally<br>3 Frequently<br>4 Often<br>5 Always
23.	How often do you find yourself saying "just a few more minutes" when online?	0 Not Applicable<br>1 Rarely<br>2 Occasionally<br>3 Frequently<br>4 Often<br>5 Always
24.	How often do you try to cut down the amount of time you spend online and fail?	0 Not Applicable<br>1 Rarely<br>2 Occasionally<br>3 Frequently<br>4 Often<br>5 Always
25.	How often do you try to hide how long you've been online?	0 Not Applicable<br>1 Rarely<br>2 Occasionally<br>3 Frequently<br>4 Often<br>5 Always
26.	How often do you choose to spend more time online over going out with others?	0 Not Applicable<br>1 Rarely<br>2 Occasionally<br>3 Frequently<br>4 Often<br>5 Always
27.	How often do you feel depressed, moody, or nervous when you are off-line, which goes away once you are back online?	0 Not Applicable<br>1 Rarely<br>2 Occasionally<br>3 Frequently<br>4 Often<br>5 Always

Comments and suggestions are highly welcomed

....

बी. पी. कोइराला स्वास्थ्य बिज्ञान प्रतिष्ठान धरान, नेपाल

सर्वेक्षण बिवरण

अनुसन्धान शिर्षक: बागमती प्रदेशका बिद्यालयहरुमा अध्ययनरत किशोर किशोरीहरूको मानसिक स्वास्थ्य स्थिति र यससँग सम्बन्धित कारकहरुको मूल्यांकन |

कोड न.: **मिति:**

बिद्यालयको नाम:

**सामाजिक-जनसांख्यिक बिवरण:**			
**सि.न.**	**प्रश्नहरु**	**प्रतिक्रिया**	**कोड न.**
1	तपाइ कति बर्ष पुरा हुनुभयो ?	…………….. बर्ष	
2	लिङ्ग	a. पुरुष<br>b. महिला<br>c. अन्य	
3	जातीय बर्गिकरण	a. दलित<br>b. जनजाती<br>c. मधेसी<br>d. मुस्लिम<br>e. ब्राह्मण / क्षेत्री<br>f. अन्य	
4	धर्म	…………………………..…	
5	स्थाई ठेगाना	…………………………..…	
6	हालको ठेगाना	…………………………..…	
7	परिवारको किसिम	**a.** एकल (nuclear)<br>**b.** संयुक्त (joint)	
8	भाइबहिनीहरुको संख्या	……………………………	
9	शैक्षिक योग्यता (कक्षा)	--------------------------------	
10	बिद्यालयको किसिम	a. सार्वजनिक / सरकारी<br>b. निजी	
11	बुवाको शैक्षिक योग्यता	....	
12	बुवाकोको पेशा	....	
13	आमाको शैक्षिक योग्यता	**………………………**......	
14	आमाको पेशा	....	
15	मासिक पारिवारिक आय	रु..	
16	के तपाईको ब्रेकअप भएको छ ?	a. छ<br>b. छैन	
17	बस्ने व्यवस्था	a. आफ्नै परिवारसंग ,<br>b. अन्य	
18	सुत्ने समय	सुत्ने समय ……..<br>उठ्ने समय ………..	
19	के तपाई आफ्नो शैक्षिक उपलब्धिमा सन्तुष्ट हुनुहुन्छ ?	a. छ	

		b. छैन	
20	शिक्षकहरुबाट कति सहयोग पाउनु भएको छ ?	a. सधैँ जसो<br>b. कहिलेकाही<br>c. कहिले पनि पाएन	
21	परिवारका शारीरिक र मानसिक रुपमा फरक क्षमता भएका कुनै सदश्य हुनुहुन्छ?	a. छ<br>b. छैन	
**आहार र व्यायाम सम्बन्धि व्यवहार:**			
22	शरीरको उचाई (cm)		
23	शरीरको तौल (kg)		
24	बिगत ३० दिनमा, घरमा प्रयाप्त खाना नहुनाले तपाई कति पटक जस्तोभोकै बस्नु पर्यो ?	a. कहिले पनि बसेन<br>b. कहिलेकाही<br>c. सधैँ जसो	
25	बिगत ७ दिनमा, तपाइले काउली, बन्दा, भन्टा, फर्सी जस्ता तरकारी दिनमा प्राय कति पटक खानुभयो ?	a. कहिले पनि खाएन<br>b. कहिलेकाही<br>c. सधैँ जसो	
26	बिगत ७ दिनमा, तपाइले स्याउ, सुन्तला, आँप वा मेवा जस्ता फलफूल दिनमा प्राय कति पटक खानुभयो ?	a. कहिले पनि खाएन<br>b. कहिलेकाही<br>c. सधैँ जसो	
27	बिगत ७ दिनमा, तपाइले जंक फुड (म:म, चाउमिन,पिज्जा, बर्गर, चाउचाउ, दलमोट, कोक फेन्टा आदि) दिनमा प्राय कति पटक खानुभयो ?	a. कहिले पनि खाएन<br>b. कहिलेकाही<br>c. सधैँ जसो	
28	बिगत ७ दिनमा प्रत्येक दिन कम्तिमा ६० मिनेटका दरले तपाई कति दिन शारीरिक रुपमा सकृय (physical activities) हुनु भयो?	a. 0 दिन<br>b. 1 दिन<br>c. 2 दिन<br>d. 3 दिन<br>e. 4 दिन<br>f. 5 दिन<br>g. 6 दिन<br>h. 7 दिन	
29	यस शैक्षिक सत्रमा (बर्षमा), के तपाईलाई कुनै कक्षामा शारीरिक क्रियाकलापको फाइदाको बारेमा पढाइयो?	a. पढाइयो<br>b. पढाइएन<br>c. मलाई थाहा भएन	
**सुरक्षात्मक तत्वहरु:**			
30	तपाइको मिल्ने साथीहरु कतिजना छन्?	a. कोहि पनि छैनन्<br>b. १ जना<br>c. २ जना<br>d. ३ वा सो भन्दा बढी	
31	तपाइले कठिन समस्या र चिन्ताहरुको बारेमा कसैसंग कति पटक कुरा गर्नु सक्नुभयो ?	a. गर्न सकेन<br>b. कहिलेकाही<br>c. धेरै जसो	
32	तपाईको परिवार वा संरक्षकले तपाईको समस्या र चिन्तालाई कत्तिको बुझ्नुहुन्छ ?	a. कहिले बुझ्नु हुन्न<br>b. कहिलेकाही	

		c. धेरै जसो	
**मदिरा (रक्सी), चुरोट र अन्य सुर्तीजन्य पदार्थ र शारीरिक (यौन) सम्पर्क सम्बन्धि:**			
33	के तपाई मदिरा (रक्सी) सेवन गर्नुहुन्छ ?	a. छ<br>b. छैन	
34	के तपाई चुरोट र अन्य सुर्तीजन्य पदार्थ सेवन गर्नुहुन्छ?	a. छ<br>b. छैन	
35	के तपाई लागु पदार्थ सेवन गर्नु हुन्छ?	a. छ<br>b. छैन	
36	यस शैक्षिक सत्रमा, के तपाइले कुनै मदिरा, चुरोट र अन्य सुर्तीजन्य पदार्थ र लागुपदार्थ सेवनले निम्त्याउने समयको बारेमा पढाइएको थियो?	a. थियो<br>b. थिएन<br>c. मलाई थाहा भएन	
37	अहिले सम्म तपाइले कतिजना संग शारीरिक (यौन) सम्पर्क गर्नुभएको छ?	a. अहुले सम्म गरेको छैन<br>b. १ जना<br>c. २ जना<br>d. २ जना भन्दा बढी	
38	शारीरिक सम्पर्कको बेला तपाई वा तपाईको यौन साथीले कण्डम वा ढालको प्रयोग गर्नुहुन्छ ?	a. कहिलेपनि प्रयोग गरेन<br>b. कहिलेकाही<br>c. सधैँ	
39	यस शैक्षिक सत्रमा, के तपाइले कुनै कक्षामा के कसरी यौन रोग, एच.आइ.भी. वा एड्स संक्रमणबाट जोगिने भन्ने बारेमा पढाइयो ?	a. पढाइयो<br>b. पढाइएन<br>c. मलाई थाहा भएन	
**चोटपटक, भौतिक आक्रमण, भौतिक झगडा र बुलिंग सम्बन्धि:**			
40	बिगत १२ महिनामा, तपाईलाई कति पटक गम्भीर रुपमा चोटपटक लागेको थियो ?	a. 0 चोटि<br>b. १ चोटि<br>c. २ वा भन्दा बढी	
41	बिगत १२ महिनामा, तपाईलाई सबैभन्दा गम्भीर रुपमा लागेको चोटपटक के थियो ?	a. बिगत १२ महिनामा मलाई त्यस्तो चोटपटक भएन<br>b. म सवारी दुर्घटनामा परेको थिए वा गाडीले हिर्काएको थियो<br>c. म लडेको थिए<br>d. म माथि केहि चिज खसेको वा केहि चिजले लागेको थियो<br>e. म माथि आक्रमण वा हिंसा भएको वा म कसैसंग झगडा गरिरहेको थिए<br>f. म आगलागीमा परेको, आगोको वा कुनै तातो चिजको नजिक थिए<br>g. मैले केहि नराम्रो (असरदार) चिज सुंघेको वा निलेको थिए<br>h. अरु केहि चिजले मलाई चोटपटक लागेको थियो	
42	बिगत १२ महिनामा तपाईमाथि कतिपटक भौतिक आक्रमण भएको थियो?	a. 0 चोटि<br>b. १ चोटि	

		c. २ वा भन्दा बढी	
43	बिगत १२ महिनामा तपाईले कतिपटक भौतिक झगडा गर्नुभएको थियो?	a. 0 चोटि<br>b. १ चोटि<br>c. २ वा भन्दा बढी	
44	तपाइले बिगत ३० दिनमा कति पटक बुलिंगको सामना गर्नुभयो?	a. कहिले गरेन<br>b. १ पटक<br>c. २ वा सो भन्दा बढी समय	
45	बुलिंग सामना गरेको स्थान	a. बिद्यालय भित्र<br>b. विद्यालय बाहिर	
46	कस्तो प्रकारको बुलिंगको सामना गर्नु भयो?	a. शारीरिक<br>b. मौखिक<br>c. सामाजिक<br>d. साइबर	
**आत्महानि र आत्मघाती व्यवहार**			
47	बिगत १२ महिनामा तपाइले जानीजानी आफैलाई कति पटक हानि गर्नुभएको छ ?	a. गरेको छैन<br>b. १ पटक<br>c. २ वा २ भन्दा बढी पटक	
48	बिगत १२ महिनामा, के तपाइले आत्महत्याको प्रयास गर्ने बारे गम्भीरतापूर्वक बिचार गर्नुभयो ?	a. थियो<br>b. थिएन	
49	बिगत १२ महिनामा, के तपाइले आत्महत्याको प्रयास (कसरि गर्ने, कुन तरिकाले गर्ने, कुन समय र कुन ठाउँमा) गर्ने भन्ने बारे योजना बनाउनु भयो |	a. थियो<br>b. थिएन	
50	पछिल्लो १२ महिनामा कति पटक आत्महत्याको प्रयास गर्नुभयो?	a. ० पटक<br>b. १ पटक<br>c. २ वा सो भन्दाबढी पटक	
51	के तपाईको नजिकको परिवारमा (आमा, बुवा, भाइ, बहिनी) कसैले आत्महत्याको प्रयास गरेको वा मृत्यु भएको छ ?	a. छ<br>b. छैन	
52	यस शैक्षिक सत्रमा, के तपाइलाई कुनै कक्षा मानसिक स्वास्थ्य स्थिति (चिन्ता, डिप्रेसनका संकेत, अनिन्द्रा र आत्महत्या) संग सम्बन्धित व्यवहार र मानसिक स्वास्थ्य सुधार कसरि गर्ने भन्ने बारे पढाएको थियो ?	a. थियो<br>b. थिएन<br>c. थाहा भएन	
**बिद्यालय स्वास्थ्य सेवा प्रदायक सम्बन्धि:**			
53	के तपाइको बिद्यालयमा स्वास्थ्यकर्मी / बिद्यालय हेल्थ नर्स सेवा उपलब्ध छ ?	a. छ<br>b. छैन	
54	तपाइको बिचारमा बिद्यार्थीहरुको स्वास्थ्य सुधारको लागि बिद्यालयमा स्वास्थ्यकर्मी/ बिद्यालय हेल्थ नर्स कतिको आवश्यक छ ?	a. धेरै<br>b. ठिकै<br>c. आवश्यक छैन	

सि.न.	**Insomnia / अनिन्द्रा**<br>**यो प्रश्नावली तपाईको पछिल्लो दुइ हप्ताको अनिन्द्राको बारेमा जान्नको लागि हो |**	
1	निदाउन कतिको गाह्रो हुन्छ ?	०. हुदैन<br>१. हल्का<br>२. ठिकै<br>३. गम्भीर<br>४. धेरै गम्भीर
2	निन्द्रा पटक पटक टुट्ने गर्छ ?	०. हुदैन<br>१. हल्का<br>२. ठिकै<br>३. गम्भीर<br>४. धेरै गम्भीर
3	निन्द्रा छिट्टै खुल्ने एंव निन्द्रा खुलेपछि निन्द्रा नलाग्ने ?	०. हुदैन<br>१. हल्का<br>२. ठिकै<br>३. गम्भीर<br>४. धेरै गम्भीर
4	तपाई अहिले आफ्नो निन्द्रासंग कतिको सन्तुष्ट हुनुहुन्छ ?	०. धेरै सन्तुष्ट छु<br>१. सन्तुष्ट<br>२. हल्का<br>३. असन्तुष्ट<br>४. धेरै असन्तुष्ट
5	तपाइको अनुसार अनिन्द्राको कारणले तपाइको दैनिक जीवनमा आउने समस्याको बारेमा तपाईको आफ्नो वरीपरीको ब्यक्तिहरुले कतिको थाहा पाउछन् ?	०. कति पनि थाहा पाउदैनन्<br>१. धेरै कम<br>२. हल्का<br>३. धेरै<br>४. एकदम धेरै
6	तपाई आफ्नो निन्द्राको समस्याको बारेमा कतिको चिन्तित हुनुहुन्छ	०. कति पनि चिन्तित छैन<br>१. धेरै कम<br>२. हल्का<br>३. धेरै<br>४. एकदम धेरै चिन्तित छु	

7	तपाइको अनुसार निन्द्राको समस्याको कारणले हजुरको दिनचर्यामा (जस्तै दिनमा थकाई लाग्ने, मन खराब हुने, ध्यान केन्द्रित गर्न नसक्ने वा गर्ने क्षमतामा कमि हुने) कतिको प्रभाव परेको छ ?	०. कति पनि छैन<br>१. धेरै कम<br>२. हल्का<br>३. धेरै<br>४. एकदम धेरै

सि.न.	**Internet addiction**<br>**तलका प्रश्नहरू तपाइको इन्टरनेट चलाउने व्यवहार संग सम्बन्धित छन् | प्रश्न पढी दिइएका बिकल्प मध्ये सबैभन्दा मिल्ने बिकल्प रोज्नुहोला |**	
8	तपाइले आफुले चाहेकोभन्दा बढी समय अनलाइन बसेको कत्तिको पाउनुहुन्छ ?	०. कत्तिपनि बस्दिन<br>१. बिरलै<br>२. कहिलेकाही<br>३. धेरै जसो<br>४. सधैँ जसो<br>५. सधैँ
9	तपाई अनलाइन बस्नके लागि घरायसी कामकाजलाई कतिको बेवास्ता गर्नुहुन्छ ?	०. कत्तिपनि गर्दिन<br>१. बिरलै<br>२. कहिलेकाही<br>३. धेरै जसो<br>४. सधैँ जसो<br>५. सधैँ
10	तपाई साथीभाइसंगको आत्मियताभन्दा बढी इन्टरनेटमा रमाउन कतिको रुचाउनु हुन्छ?	०. कत्तिपनि रुचाउदैन<br>१. बिरलै<br>२. कहिलेकाही<br>३. धेरै जसो<br>४. सधैँ जसो<br>५. सधैँ
11	अन्य इन्टरनेट प्रयोगकर्ताहरुसंग तपाइले नयाँ सम्बन्धकतिको बनाउनुहुन्छ ?	०. कत्तिपनि बनाउदिन<br>१. बिरलै<br>२. कहिलेकाही<br>३. धेरै जसो<br>४. सधैँ जसो<br>५. सधैँ
12	तपाइले अनलाइन रहेर बिताउने समयबारे तपाईको जीवनमा रहेका अन्य व्यक्तिहरुले कत्तिको गुनासो गर्छन ?	०. कत्तिपनि गर्दैनन्<br>१. बिरलै<br>२. कहिलेकाही<br>३. धेरै जसो<br>४. सधैँ जसो<br>५. सधैँ

13	तपाइले इन्टरनेटमा अनलाइन रहेर बिताउने समयले तपाइको अध्ययनलाई कत्तिको असर पारेको छ?	०. कत्तिपनि गरेको छैन<br>१. बिरलै<br>२. कहिलेकाही<br>३. धेरै जसो<br>४. सधैँ जसो<br>५. सधैँ
14	तपाइले केहि जरुरि काम गर्नु अघि इमेल/ अनलाइन म्यासेज कत्तिको हेर्नुहुन्छ ?	०. कत्तिपनि हेर्दिन<br>१. बिरलै<br>२. कहिलेकाही<br>३. धेरै जसो<br>४. सधैँ जसो<br>५. सधैँ
15	इन्टरनेटले गर्दा तपाईको जागिर वा कार्यक्षमतालाई कतिको असर परेको छ?	०. कत्तिपनि छैन<br>१. बिरलै<br>२. कहिलेकाही<br>३. धेरै जसो<br>४. सधैँ जसो<br>५. सधैँ
16	तपाईले इन्टरनेटमा वा अनलाइन रहेर गर्ने कामको बारे कसैले सोध्दा कुरा लुकाउने वा आनाकानी गर्ने कतिको गर्नुहुन्छ ?	०. कत्तिपनि गर्दिन<br>१. बिरलै<br>२. कहिलेकाही<br>३. धेरै जसो<br>४. सधैँ जसो<br>५. सधैँ
17	आफ्नो जिन्दगीको बारेमा आउने सोचरुलाई तपाइले इन्टरनेटका रमाइला सोचहरुले कत्तिको दबाउन खोज्नुहुन्छ ?	०. कत्तिपनि खोज्दिन<br>१. बिरलै<br>२. कहिलेकाही<br>३. धेरै जसो<br>४. सधैँ जसो<br>५. सधैँ
18	तपाई अब कति समय पछि अनलाइन बस्ने भन्ने अपेक्षा गर्दै कत्तिको बस्नुहुन्छ ?	०. कत्तिपनि बस्दिन<br>१. बिरलै<br>२. कहिलेकाही<br>३. धेरै जसो<br>४. सधैँ जसो<br>५. सधैँ
19	इन्टरनेट बिनाको जिन्दगी निरस र शून्य हुने डर कत्तिको महसुस गर्नुहुन्छ ?	०. कत्तिपनि गर्दिन<br>१. बिरलै<br>२. कहिलेकाही<br>३. धेरै जसो<br>४. सधैँ जसो<br>५. सधैँ

20	अनलाइन रहँदा कसैले तपाईलाई बाधा दियो भने चिच्याउने, झिंजो माने वा गालि गर्ने कत्तिको गर्नुहुन्छ ?	०. कत्तिपनि गर्दिन<br>१. बिरलै<br>२. कहिलेकाही<br>३. धेरै जसो<br>४. सधैँ जसो<br>५. सधैँ
21	राति अबेरसम्म अनलाइन रहेर तपाईलाई निन्द्रा नै नलाग्ने कत्तिको हुन्छ ?	०. कत्तिपनि हुदैंन<br>१. बिरलै<br>२. कहिलेकाही<br>३. धेरै जसो<br>४. सधैँ जसो<br>५. सधैँ
22	तपाईलाई इन्टरनेट नचलाउँदा पनि इन्टरनेट सम्बन्धि सोचहरु वा अनलाइन रहने चाहना कत्तिको आउँछ ?	०. कत्तिपनि आउँदैन<br>१. बिरलै<br>२. कहिलेकाही<br>३. धेरै जसो<br>४. सधैँ जसो<br>५. सधैँ
23	तपाई आफुलाई अनलाइन रहँदा "अझै केहि मिनेट मात्र चलाउछु" कत्तिको भन्नुहुन्छ ?	०. कत्तिपनि भन्दिन<br>१. बिरलै<br>२. कहिलेकाही<br>३. धेरै जसो<br>४. सधैँ जसो<br>५. सधैँ
24	तपाइले अनलाइन बिताउने समय घटाउन प्रयास गर्ने तर असफल हुने कत्तिको भएको छ ?	०. कत्तिपनि छैन<br>१. बिरलै<br>२. कहिलेकाही<br>३. धेरै जसो<br>४. सधैँ जसो<br>५. सधैँ
25	तपाई आफु कति समयदेखि अनलाइन हुनुहुन्छ भन्ने कुरा लुकाउने प्रयास कत्तिको गर्नुहुन्छ ?	०. कत्तिपनि लुकाउदिन<br>१. बिरलै<br>२. कहिलेकाही<br>३. धेरै जसो<br>४. सधैँ जसो<br>५. सधैँ
26	तपाई अरुसंग बाहिर घुम्न जानुको सट्टा अनलाइन बस्ननै रुचाउने कत्तिको गर्नुहुन्छ ?	०. कत्तिपनि रुचाउदीन<br>१. बिरलै<br>२. कहिलेकाही<br>३. धेरै जसो<br>४. सधैँ जसो<br>५. सधैँ

27	तपाईलाई अफलाइन रहँदा उदास वा चंचल हुने तर अनलाइन आउने बित्तिकै सामान्य हुने कत्तिको महसुस हुन्छ ?	०. कत्तिपनि गर्दिन<br>१. बिरलै<br>२. कहिलेकाही<br>३. धेरै जसो<br>४. सधैँ जसो<br>५. सधैँ

कुनै सुझाब वा प्रतिक्रिया भए यहाँ लेख्नु होला

……

……

………………………………………………………………………………………
